# *NCAPG* Dynamically Coordinates the Myogenesis of Fetal Bovine Tissue by Adjusting Chromatin Accessibility

**DOI:** 10.3390/ijms21041248

**Published:** 2020-02-13

**Authors:** Xin Hu, Yishen Xing, Xing Fu, Qiyuan Yang, Ling Ren, Yahui Wang, Qian Li, Junya Li, Lupei Zhang

**Affiliations:** 1 Key Laboratory of Animal Genetics Breeding and Reproduction, Ministry of Agriculture and Rural Affairs; Institute of Animal Sciences, Chinese Academy of Agricultural Sciences, Beijing 100193, China; huxin19890803@163.com (X.H.); yishen_xing@163.com (Y.X.); renling5454@163.com (L.R.); wang1434243198@163.com (Y.W.); lq798711247@163.com (Q.L.); 2 Molecular and Cellular Biology, Gembloux Agro-Bio Tech, University of Liège, 5030 Gembloux, Belgium; 3 School of Animal Sciences, Louisiana State University Agricultural Center, Baton Rouge, LA 70803, USA; XFu1@agcenter.lsu.edu; 4 Department of Molecular, Cell and Cancer Biology, University of Massachusetts Medical School, Worcester, MA 01655, USA; qiyuan.yang@umassmed.edu

**Keywords:** *NCAPG*, bovine myoblasts, myogenesis, chromatin accessibility, AP-1

## Abstract

NCAPG is a subunit of condensin I that plays a crucial role in chromatin condensation during mitosis. *NCAPG* has been demonstrated to be associated with farm animal growth traits. However, its role in regulating myoblast differentiation is still unclear. We used myoblasts derived from fetal bovine tissue as an in vitro model and found that *NCAPG* was expressed during myogenic differentiation in the cytoplasm and nucleus. Silencing *NCAPG* prolonged the mitosis and impaired the differentiation due to increased myoblast apoptosis. After 1.5 days of differentiation, silencing *NCAPG* enhanced muscle-specific gene expression. An assay for transposase-accessible chromatin- high throughput sequencing (ATAC-seq) revealed that silencing *NCAPG* altered chromatin accessibility to activating protein 1 (AP-1) and its subunits. Knocking down the expression of the AP-1 subunits fos-related antigen 2 (*FOSL2)* or junB proto-oncogene *(JUNB)* enhanced part of the muscle-specific gene expression. In conclusion, our data provide valuable evidence about *NCAPG*’s function in myogenesis, as well as its potential role in gene expression.

## 1. Introduction

The growth rate is the most important trait in farm animal production. Genome variations regulating animal growth and development have been widely studied in farm animals. *NCAPG* is a gene well-documented to be associated with body size, average daily gain, feed intake efficiency, fetal growth, carcass trait, and muscle development in beef cattle [1,2,3,4,5,6,7]; body weight in sheep [8]; and withers height in horses [9,10]. Moreover, in clinical practice, *NCAPG* is a crucial gene that is correlated with height [11].

NCAPG is a subunit of the condensin I complex, which is essential for the structural maintenance of chromosomes [12]. The core subunits of condensin are *SMC2* and *SMC4*, both of which are the members of structural maintenance of chromosome (SMC) family of chromosomal ATPases [13]. Condensin I also has unique non-SMC subunits: *CAP-G*, *CAP-H*, and *CAP-D2* [12]. Previous studies have revealed that both condensin members play essential but distinct roles in mitosis. Condensin I is sequestered in the cytoplasm during the interphase and gains access to chromosomes only after the nuclear envelope break down (NEBD) in the prometaphase. Condensin II functions in the nucleus from the interphase through the prophase as an early regulator of chromosome condensation. After NEBD, condensin I engages in further chromosome compaction to facilitate final mitosis [14,15,16]. In one study, the abrogation of the *CAP-D2* subunit resulted in a significant delay of prometaphase to anaphase progression compared to the control group [15]. This provided direct evidence for the relationship between *NCAPG* and growth traits.

Skeletal muscle development is one of the most important traits in farm animal production because skeletal muscle account for about half of an animal’s body weight. Muscle development is classified into prenatal and postnatal stages. The fetal stage is crucial for skeletal muscle development, as the number of muscle fibers barely increases after birth [17,18,19].

However, the regulatory mechanisms and biological functions of *NCAPG* are still unknown. In particular, *NCAPG*’s role in the development of myoblasts in the fetal stage needs exploration. In this study, the role of *NCAPG* in the myogenic differentiation of myoblasts derived from fetal bovine tissue was determined using RNA interference to knock down *NCAPG* gene expression. Moreover, due to *NCAPG*’s conserved roles in other tissues, our study and its meanings may also be applied elsewhere in the body.

## 2. Results

### 2.1. Myogenic Differentiation of Myoblasts Derived from Fetal Bovine Tissue

**Figure 1:**
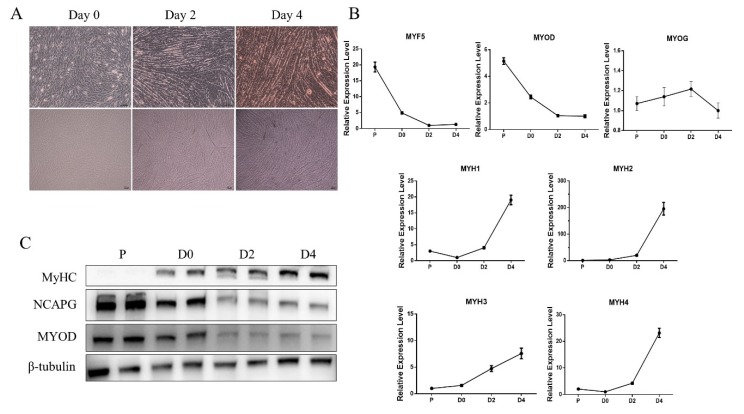
Myogenic differentiation of myoblasts derived from fetal bovine tissue. (**A**) Microscopic images of bovine myoblasts on days 0, 2, and 4 of differentiation. Scale bars = 100 µm. (**B**) Transcript levels of *MYF5, MYOD, MYOG, MYH1, MYH2, MYH3* and *MYH4* during proliferation (P) and myogenic differentiation (D0, D2, and D4). The results are represented as the mean ± the SEM from at least three independent experiments. (**C**) Western blot evaluating the protein levels of MYOD and MHCs in the cultured cells, as described in (**B**).

After 24 h of differentiation, some myotubes could be observed. After 48 h, a majority of the myoblasts had fused into myotubes. After an additional two days of differentiation, both the size and length of the myotubes increased (Figure 1A). RNA and proteins were extracted from the cells on days 0, 2, and 4 to analyze the muscle-specific gene expression profile during proliferation and differentiation. Myoblast determination protein 1 (*MYOD*) and myogenic Factor 5 (*MYF5*) mRNA expression decreased during proliferation and differentiation, while *MYOG* expression increased until day 2 of differentiation, after which it decreased (Figure 1B). 

As for myosin heavy chain (*MYH*) isoforms in skeletal muscle, the expression of *MYH1, MYH2, MYH3,* and *MYH4* was relatively low during proliferation and early differentiation, but it increased dramatically during late differentiation (Figure 1B). In particular, *MYH2* expression increased nearly 200-fold compared to basal level on day 4. Consistently, western bolt analyses showed that MYH expression increased gradually during the differentiation of myoblasts, while MYOD expression decreased during differentiation (Figure 1C). From these results, it is clear that the use of myoblasts derived from fetal tissue is a reliable model for studying early myogenic differentiation in cattle.

### 2.2. NCAPG Inhibition Prolonged the Prometaphase and Metaphase of the Proliferating Myoblasts

To investigate the role of *NCAPG*, we first analyzed its expression (as well as that of other subunit genes of condensin I and II) during proliferation and differentiation. *NCAPG* was robustly expressed during proliferation and the beginning of differentiation. The mRNA condensin core subunits *SMC2* and *SMC4* had similar expression patterns, as did the non-SMC regulatory subunits *NCAPG, NCAPH, NCAPD2, NCAPG2, NCAPH2*, and *NCAPD3*. These genes had robust expression during proliferation, but then the mRNA level attenuated until day 2. After day 2 of differentiation, their expression increased slightly (Figure S1).

**Figure 2:**
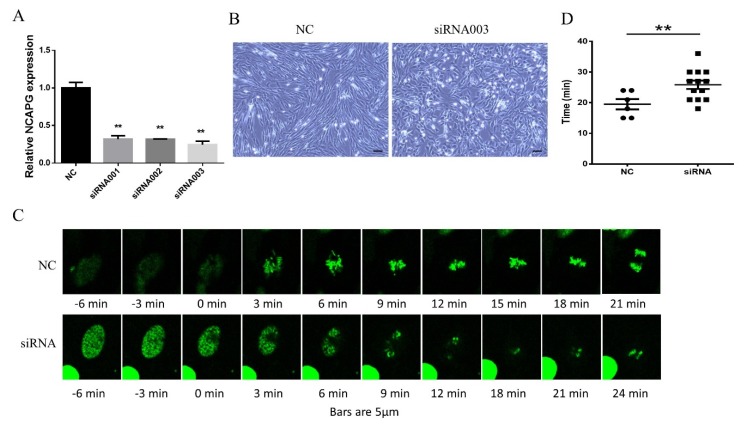
*NCAPG* KD elongated the prometaphase and metaphase. (**A**) The silencing efficiency of *NCAPG* siRNAs. We designed three siRNAs that were transfected into myoblasts, and the mRNA level was measured to determine the siRNA efficiency. The results are represented as the mean ± the SEM (three biological replicates), ** *p* < 0.01. (**B**) Cell morphology of *NCAPG* silencing myoblasts. *NCAPG* was depleted in the proliferating myoblasts. Scale bar = 100 µm. (**C**) An analysis of chromosome condensation during the prophase in live myoblasts depleted of NCAPG. To visualize chromatin, we stably expressed H2B–EGFP in the myoblasts. Prophase image sequences were extracted from long-term imaging experiments and aligned along a time axis according to nuclear envelope breakdown (NEBD). Imaging was done 48–72 h after siRNA transfection. Bar = 5 µm. (**D**) The time duration of the prometaphase and metaphase in the myoblasts. The time from NEBD to the onset of the anaphase was measured using at least six replicates, ** *p* < 0.01.

The regulatory mechanism of *NCAPG* in mitosis has previously been studied in rodent cells. To explore its function in bovine myoblasts, we designed three siRNAs targeting common exons of *NCAPG* transcript isoforms to knock down total *NCAPG* mRNA. Here, siRNA003 was selected for the knockdown experiments due to its high efficiency (Figure 2A). We first identified a morphological change in the myoblasts in the knowndown (KD) group, which appeared as a flattened stellate shape (Figure 2B). 

To confirm *NCAPG*’s role in bovine myoblast mitosis, we employed a lentivirus to express the fused bH2B–EGFP gene while knocking down *NCAPG* mRNA expression. About one day after the lentivirus infection, green fluorescent protein (GFP) expression was clustered in the nucleus. Then, the myoblasts were subcultured at a low density and transfected with siRNA. In order to observe the mitotic process, pictures were taken every 3 min during proliferation for two days after siRNA transfection (Figure 2C). The time between NEBD and the onset of the anaphase was determined. The siRNA control (NC) group required about 19.5 ± 4.1 min from NEBD to initiate of anaphase, whereas the siRNA group took about 25.8 ± 5.0 min (Figure 2D). Therefore, we found that the knockdown of *NCAPG* prolonged the procession of myoblast mitosis.

### 2.3. NCAPG Inhibition Impaired Myogenic Differentiation in Myoblasts

Until this study, the role of *NCAPG* in myogenic differentiation had not been explored. Intriguingly, during differentiation, both the mRNA and protein expression of *NCAPG* were relatively low compared to during proliferation (Figure 1C and Figure S1). First, we detected *NCAPG* localization in the myoblasts. The immunofluorescence (IF) results showed that NCAPG could be detected in both the nucleus and cytoplasm of the myoblasts on day 2 of differentiation (Figure S2A). Subsequently, in order to explore the distribution of *NCAPG* in the myoblasts, we analyzed its presence in the cytoplasm and nucleus (separately). Here, β-tubulin and H2B were used as a reference for cytoplasm proteins and nuclear proteins, respectively. The immunostaining results showed that NCAPG was localized in both the cytoplasm and nucleus (Figure S2B). This nuclear localization made it possible for *NCAPG* to access chromatin. This suggests that *NCAPG* took part in regulating myogenic differentiation. 

**Figure 3:**
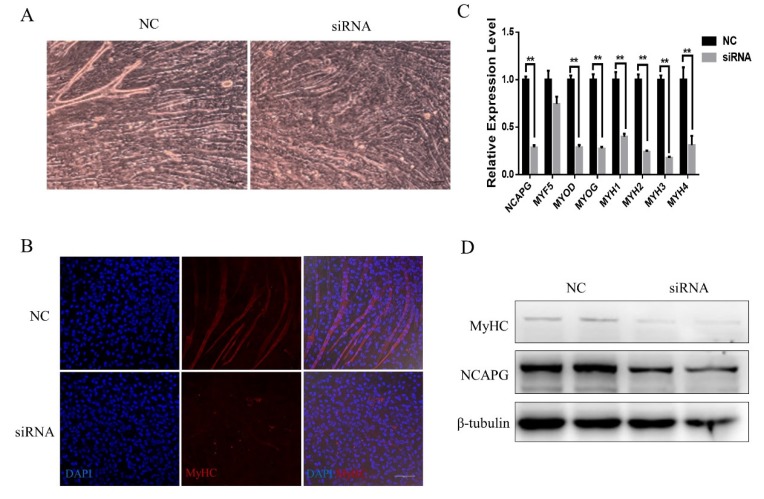
Silencing NCAPG impaired the differentiation of myoblasts. (**A**) Cell morphology of NCAPG-silenced myoblasts on day 4 of differentiation. Scale bar = 100 µm. (**B**) NCAPG-silenced myoblasts on day 4 of differentiation. Myotubes were visualized using IF staining with MYH antibodies. Scale bar = 100 µm. (**C**) Transcript levels of muscle-specific genes on day 4 of differentiation. The results are presented as the mean ± the SEM from at least three independent experiments: ** *p* < 0.01. (**D**) Western blot evaluating the protein levels of NCAPG and MHCs in myoblasts on day 4 of differentiation.

To investigate the role of *NCAPG* in myogenic differentiation, we interfered with its expression by transfecting siRNA003, while scrambled siRNA used as a control. Myotubes appeared around 48 h after differentiation. About four days after the induction of myogenesis, we could observe obvious myotubes in the NC group, whereas KD caused dysfunctional fusion (Figure 3A). Then, an IF test was done using an MYH antibody to identify the myotubes, and a similar difference was observed (Figure 3B). To further explore the alteration in gene expression, we extracted RNA and proteins from the myoblasts four days after *NCAPG* knockdown. *NCAPG* expression had decreased by about 70% by day 4. We detected the expression of some muscle-specific genes. For instance, *MYH1*, *MYH2*, *MYH3*, *MYH4*, *MYOD*, and *MYOG* expression was significantly decreased in the KD group (*p* < 0.01, Figure 3C). However, a difference in *MYF5* expression between the two groups was not detected (Figure 3C). Immunostaining showed similar results: the protein levels of MYHs and NCAPG decreased after *NCAPG* knockdown (Figure 3D).

### 2.4. NCAPG KD Facilitated Apoptosis

**Figure 4:**
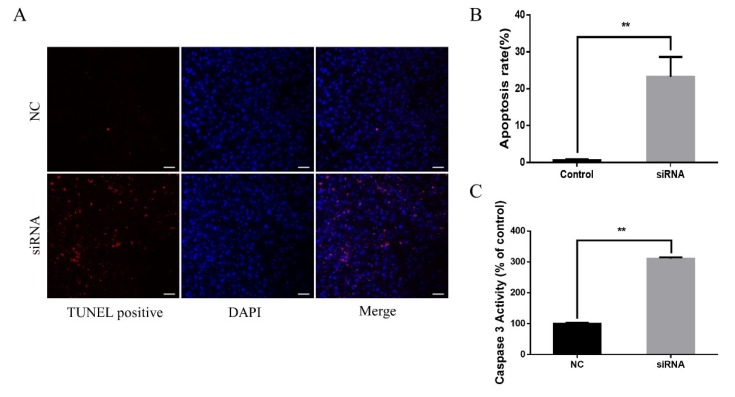
*NCAPG* KD induced apoptosis. (**A**) Transcript levels of muscle-specific gene expression in AP-1 subunits (FOSL2) in depleted myoblasts. (**B**,**C**) Transcript levels of muscle-specific gene expression in AP-1 subunits (JUNB) in depleted myoblasts. The results are presented as the mean ± the SEM from at least three independent experiments, * *p* < 0.05, ** *p* < 0.01.

To explore the mechanism of *NCAPG* in impairing myogenesis, we detected apoptosis on day 4 using a TdT-mediated dUTP nick-end labeling (TUNEL) assay and a caspase 3 activity assay. As shown in Figure 4A, *NCAPG* knockdown increased the number of TUNEL-positive cells on day 4. About 23% of cells in the siRNA group were TUNEL-positive, while the apoptosis rate of the NC group was less than 1% (Figure 4B). The caspase 3 activity assay showed that *NCAPG* knockdown led to a three-fold increase in caspase-3 activity compared to the NC group (Figure 4C).

### 2.5. Myogenic Gene Expression was Boosted on Day 1.5 of Differentiation after NCAPG Knockdown

**Figure 5:**
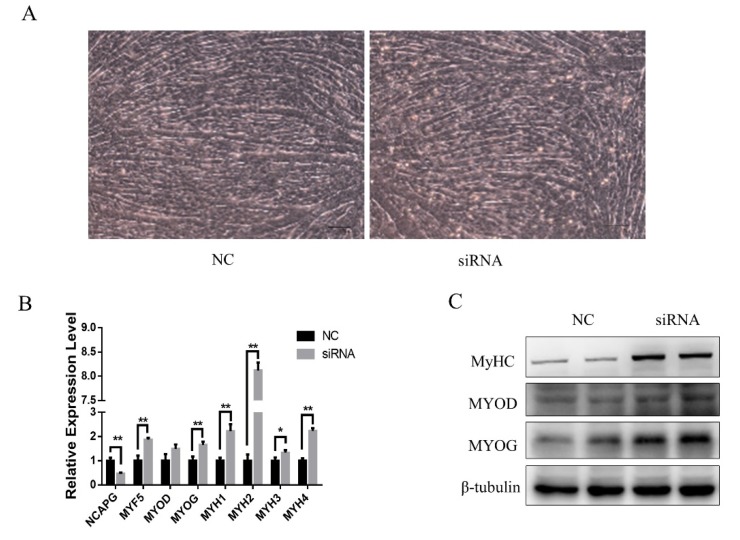
Myogenic gene expression was upregulated after 1.5 d of differentiation in NCAPG-silenced myoblast. (**A**) The cell morphology of *NCAPG*-silenced myoblasts on day 1.5 of differentiation. Scale bar = 100 µm. (**B**) Transcript levels of muscle-specific genes on day 1.5 of differentiation. The results are presented as the mean ± the SEM from at least three independent experiments: * *p* < 0.05, ** *p* < 0.01. (**C**) Western blot evaluating the protein levels of MYOD, MYOG, and MHCs in myoblasts on day 1.5 of differentiation.

Interestingly, despite the reduced myogenesis in the *NCAPG* knockdown myoblasts after four days of myogenesis, we found that the myotube formation of the siRNA group was slightly higher than that of the NC group at 1.5–2 days of myogenesis (Figure 5A). At 36 h, we analyzed the muscle-specific gene expression as well as the condensin subunit genes. The results showed that in the siRNA group, the mRNA levels of *MYH1*, *MYH2*, *MYH3*, *MYH4*, *MYF5*, and *MYOG* were significantly higher than in the NC group. There was no difference in the expression of *MYOD* between groups (Figure 5B). Consistently, the protein expression analysis showed that the expression of MYHs and MYOG in the siRNA group was upregulated, while MYOD was not affected (Figure 5C).

### 2.6. NCAPG Knockdown Altered Chromatin Compaction and Accessibility

**Figure 6:**
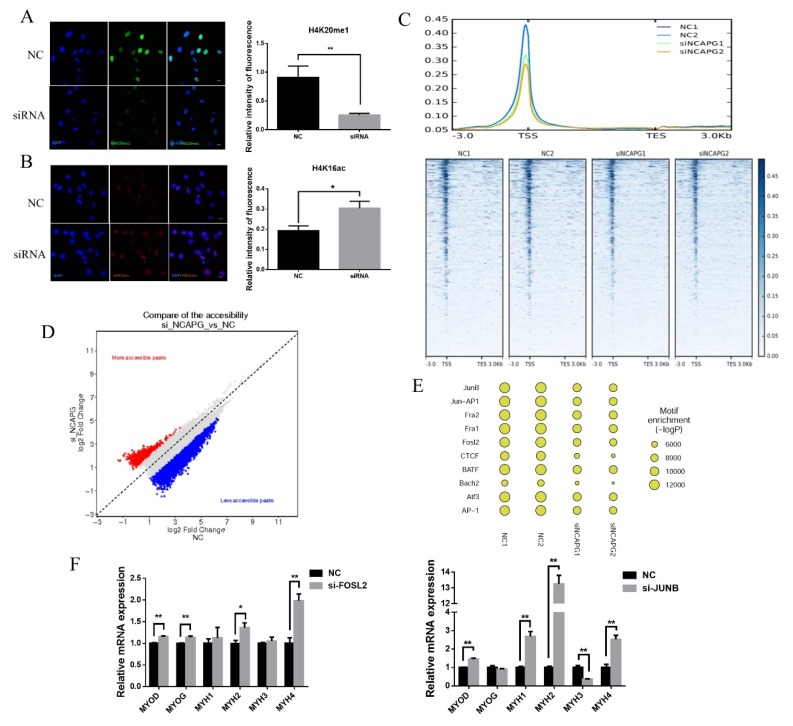
NCAPG knockdown altered chromatin compaction and accessibility. (**A**,**B**) (Left) Representative immunofluorescence images of myoblasts transfected with a negative control or si-NCAPG and stained with antibodies against H4K20me1 (green) or H4K16ac (red). DNA (DAPI) is shown in blue. Scale bars = 10 μm. (Right) Fluorescence intensity quantification. The results are presented as the mean ± the SEM, * *p* < 0.05, ** *p* < 0.01. (**C**) (**top**) A histogram of the intensity of enriched reads (from TSS −3 kb to TES +3 kb) for each replicate. (**bottom**) A heatmap of ATAC-seq signal mapping with annotated TSS. (**D**) Scatterplots showing changes in chromatin accessibility between the NC and si-NCAPG groups. (**E**) TF motifs identified from ATAC-seq peaks. (**F**) (**Left**) Transcript levels of muscle-specific gene expression in AP-1 subunits (FOSL2) in depleted myoblasts. (**Right**) Transcript levels of muscle-specific gene expression in AP-1 subunits (JUNB) in depleted myoblasts. The results are presented as the mean ± the SEM from at least three independent experiments, * *p* < 0.05, ** *p* < 0.01.

To investigate the relationship between altered gene expression and *NCAPG* knockdown, we conducted an IF test using H4K20me1 and H4K16ac antibodies. H4K20me1 modification is positively correlated with chromatin compaction, while H4K16ac is negatively correlated with chromatin compaction [20,21]. The results showed that the fluorescence intensity of H4K20me1 in the siRNA group attenuated significantly, while that of H4K16ac increased significantly after knockdown on day 1.5 (Figure 6A,B). These results demonstrated that *NCAPG* knockdown reduced chromatin compaction. To further study chromatin compaction after *NCAPG* knockdown, we used an ATAC-seq to isolate the accessible chromatin on day 1.5. The sequencing reads were mapped to the bovine genome, and summarized statistics of the read mapping are listed in Table S1. The reads from both the NC and si-*NCAPG* groups were enriched around transcription start sites (TSS) (Figure 6C), and the peaks were called using MACS2. The peak number from the NC group was around 110,000, while the peak number from the si-NCAPG group varied between 71,000 and 75,000 (Table S2). The annotation of peaks revealed that silencing *NCAPG* resulted in the proportion of the peaks located in −1000 bp (Figure S3). However, compared to NC, silencing NCAPG led to more of a decrease in the accessibility of chromatin sites (Figure 6D). Pearson correlation coefficients between the NC and si-*NCAPG* groups were calculated based on the Log_10_ RPM matrix (Figure S4). A Gene Ontology analysis revealed that silencing NCAPG affected multiple processes, including development, cell differentiation, and the positive regulation of biological processes (Figure S5). The Kyoto Encyclopedia of Genes and Genomes (KEGG) analysis also revealed the pathways regulated by *NCAPG*, including the metabolic pathway, the MAPK pathway, the regulation of actin cytoskeleton pathway, and the calcium signaling pathway (Figure S5). The metabolic pathway had the highest proportion of genes with altered accessibility, including PRKAA1 and SIRT1 (Figure S6). The 10 most enriched motifs are listed in Figure 6E. A motif analysis revealed that silencing *NCAPG* induced the decreased accessibility of chromatin fragments containing transcription factors (TF) binding sites such as AP-1 subunits (JUNB, FOSL2, and FOSL1), CCCTC-binding factor (CTCF), and basic leucine zipper ATF-Like transcription factor (BATF) (Figure 6E).

To analyze the relationship between AP-1 binding and muscle-specific gene expression, we knocked down the AP-1 subunit genes FOSL2 and JUNB using siRNA. FOSL2 silencing increased the expression of *MYOD*, *MYOG*, *MYH2*, and *MYH4*, while *JUNB* silencing improved the expression of *MYOD*, *MYH1*, *MYH2*, and *MYH4* but repressed *MYH3* expression (Figure 6F).

## 3. Discussion

The location of condensins in the cytoplasm and their role in mitosis have been intensively investigated [14,15,16]. Condensin II primarily contributes to the axial shortening of chromatids, while condensin I has a role in lateral compaction [22]. In one study, depletion of the condensin I subunit prolonged prometaphase-to-anaphase progression by more than 150% [15]. In our study, we found a similar delay after silencing *NCAPG*. However, prometaphase-to-anaphase progress was delayed by about 32% compared to the control group. The difference may have been due to the cell type, the siRNA concentration and efficiency, and the condensin subunit selected.

The common view of condensin localization is that condensin I is cytoplasmic during the interphase and, after the prophase and NEBD, has access to chromatin [15,23]. However, recent reports have indicated that a small amount of condensin I can be found in the nucleus during the G1 phase, which is gradually lost during the S and G2 phases [24]. In those reports, silencing the subunits of condensin I in the G0/G1-phase cells resulted in gene misregulation [24,25]. In our study, all condensin subunits, including *NCAPG*, decreased dramatically after the initiation of myogenic differentiation and remained at a relatively low level late in the differentiation process. We also found *NCAPG* expressed in the nuclei of differentiating myogenic myoblast cells. This implies that condensin I may function not only during mitosis, but also during other stages of the cell cycle.

We found that knocking down *NCAPG* during the myogenic differentiation of fetal bovine muscle myoblasts promoted apoptosis. The association between apoptosis and *NCAPG* or condensin I depletion has been reported in several studies. In zebrafish, a mutation in *NCAPG* resulted in increased genomic imbalances and in an increased rate of apoptosis in the retina [26]. In another work, silencing *NCAPG* in hepatocellular carcinoma cells inhibited proliferation and induced apoptosis [27,28]. Condensin I disruption has also led to apoptosis in the germline [29]. Further, knocking down *NCAPD2*, a subunit of condensin I, has led to apoptosis in triple-negative breast cancer cells [30].

In contrast to its established role in chromatin condensation in mitosis, our knowledge about condensin I in regulating gene expression is still limited. Condensin I is largely absent from heterochromatic regions and binds predominantly to promoter sequences of active genes in mitotic chicken DT40 cells [31]. In human cell mitosis, TATA-binding protein (TBP) transmits active gene memory to daughter cells by directly interacting with *NCAPG* in the vicinity of these promoters via its associated protein phosphatase 2A (PP2A), thereby inhibiting the compaction of these regions [32]. In *Drosophila melanogaster*, condensin I has been implicated in the repression of homeotic genes [33]. In yeast, Hocquet et al. have reported that condensins play no direct role in the maintenance of the transcriptome either during interphase or during mitosis: in that study, the gene expression changes in postmitotic fission yeast cells were largely a consequence of chromosome mis-segregation during the anaphase [34]. Condensin depletion causes genome decompaction without altering the level of global gene expression in *saccharomyces cerevisiae* [35]. In one study, condensin II physically interacted with TFIIIC, and they both colocalized at active gene promoters (in mouse and human genomes): this was facilitated by the interaction between NCAPD3 and the epigenetic mark H3K4me3 [36]. Thus far, previous studies have not provided conclusive evidence showing how condensin I regulates gene expression. In our study, chromatin condensation decreased after *NCAPG* silencing. We also found that muscle-specific genes were first upregulated after the silencing of *NCAPG*, but then they decreased. Because accessibility is related to chromatin condensation [37] and histone acetylation [38], we suspect that *NCAPG* might affect chromatin accessibility.

Using an ATAC-seq and motif analysis, we found that AP-1 enrichment (and that of its subunits) at accessible chromatin regions decreased after *NCAPG* silencing. AP-1 transcription is a dimeric complex involved in diverse cellular processes such as proliferation, differentiation, and apoptosis [39]. This dimeric complex is composed of *Jun* (*c-Jun*, *JunB*, and *JunD*) and *Fos* (*c-Fos*, *FosB*, *Fra-1*, and *Fra-2*) proto-oncogene proteins that bind to a cis-element called the TRE (12-O-tetradecanoylphorbol-13-acetate response element) [39]. Different AP-1 dimer combinations are crucial for AP-1 binding activities and their biological function [40,41,42]. In one study of skeletal muscle cells, AP-1 binding sites were also enriched in a large subset of MyoD-regulated genes, many of which were downregulated during differentiation [43]. During myogenic differentiation, Fra-2 is a major component of the AP-1 complex in differentiating cells [44]. For instance, silencing Fra-2 increases the protein expression of terminal differentiation markers such as muscle creatine kinase (MCK) and *MYH*C [45]. Previous research has reported that *c-Jun* can inhibit differentiation by directly binding to *MYOD* [46]. *MYOD* can act as a negative regulator in *c-fos* transcription by binding with serum-responsive elements in the c-fos promoter [47]. *JunB* is also involved in the early steps of the inhibition of myogenic differentiation. An increase in *JunB* mRNA expression is highly correlated with AP-1 binding activities and inhibits the expression of myoblast differentiation markers in C2C12 cells [48]. Here, we used siRNA to silence the AP-1 subunits *JunB* and *Fra-2* and found increased expression in most muscle-specific genes.

In conclusion, *NCAPG* is indispensable in myogenic differentiation, and the lack of *NCAPG* induces apoptosis. *NCAPG* also regulates chromatin accessibility to AP-1, a ubiquitously expressed TF complex, and subsequently affects gene expression. These results provide new evidence for NCAPG’s function in myogenesis, as well as its potential role in gene expression.

## 4. Materials and Methods

### 4.1. Ethics Statement

The animal experiments were performed according to the guidelines established by the Regulations for the Administration of Affairs Concerning Experimental Animals (Ministry of Science and Technology, China, 2004). All animal experimental protocols in this study were carried out in strict conformance with the rules of the Animal Ethics Committee of the Institute of Animal Sciences, Chinese Academy of Agricultural Sciences (No. IAS2019-48; 12/9/2019). Pregnant cows were raised by the Inner Mongolia Aokesi Agriculture Co., Ltd. (Wulagai, China). All efforts were made to minimize the cows’ suffering.

### 4.2. Primary Cell Isolation and Cell Culture

The myoblast cells were enzymatically isolated from longissimus dorsi tissues obtained from bovine fetuses 90 to 120 days old, as described previously [49]. At 70%–80% confluence, the cells were passaged with 0.25% trypsin-EDTA (Gibco, Grand Island, NY, USA). After reaching 100% confluence, cells were induced in differentiation medium (DM) consisting of DMEM containing 5% horse serum (Gibco). The medium was exchanged every 2 days.

### 4.3. siRNA Transfection and Gene Knockdown

The transfection of siRNA into myoblast cells was performed using Lipofectamine™ RNAiMAX transfection reagent (Invitrogen Life Technologies, Carlsbad, CA, USA) when the cells reached 100% confluence.

Small interfering RNA (siRNA) was obtained from RiboBio (Guangzhou, China), and the sequences are shown in Table S3: siRNA transfection was performed by following the manufacturer′s recommended procedure. Cells were transfected with siRNA against *NCAPG* (siNCAPG) or siRNA control (NC) at a final concentration of 50 nM.

### 4.4. RNA Extraction, cDNA Synthesis, and Quantitative Real-Time PCR

Total RNA was extracted from cells using TRIzol reagent (Invitrogen Life Technologies). RNA concentration and quality were assessed by a NanoPhotometer N50 (Implen, Munich, Germany) and 1.5% agarose gel electrophoresis. A cDNA synthesis for mRNA was performed using PrimeScript RT Master Mix (Perfect Real Time) (TaKaRa, Kusatsu, Japan). In addition, qRT-PCR was performed on a QuantStudio 7 Flex Real-Time PCR System (Life, Carlsbad, CA, USA) with a KAPA SYBR^®^ FAST qPCR Kit (KAPABiosystems, Wilmington, MA, USA). Samples from at least three independent experiments were assayed following the manufacturer′s instructions. The sequence of qRT-PCR detection primers can be found in CS4.

### 4.5. Immunofluorescence

The immunofluorescence tests were performed in myoblast cells cultured in 12-well plates. Cells were fixed in 4% paraformaldehyde for 15 min and then washed three times with phosphate-buffered saline (PBS). Subsequently, cells were incubated in 0.1% Triton X-100 diluted by PBS for 10 min at room temperature, and the cells were blocked with 1% albumin bovine serum (Beyotime, Shanghai, China) for 30 min in order to reduce the nonspecific binding of primary antibodies. After incubation with the primary antibodies overnight at 4 °C, secondary antibodies were added, and the cells were incubated at room temperature for 1 h. The cell nuclei were stained with 4′,6-diamidino-2-phenylindole (DAPI) (Sigma-Aldrich, St. Louis, MO, USA) for 5 min, and images were obtained with a confocal microscope (TCS SP8, Leica, Wetzlar, Germany). The following antibodies were used: MHC antibody (MF20, Developmental Studies Hybridoma Bank, Iowa, USA, 1:100); NCAPG (sc-101014, Santa Cruz, Delaware Ave Santa Cruz, CA, USA, 1:1000); NCAPD3 (16828-1-AP, Proteintech, Chicago, IL, USA, 1:500); H4K20me1 (ab9051, Abcam, Cambridge, UK, 1:1000); and H4K16ac (13534, Cell Signaling Technology, Danvers, MA, USA, 1:1600).

### 4.6. Western Blot

Cells were digested with low-concentration trypsin and collected into tubes. We used a Nuclear and Cytoplasm Protein Extraction Kit (Beyotime) to separate nuclear and cytoplasm proteins. Cells were lysed in an ice cold radio immunoprecipitation assay (RIPA) lysis buffer with 1 mM phenylmethyl sulfonyl fluoride (Sigma-Aldrich), which was used for cell protein extraction. Protein concentration was determined using a BCA KIT (Beyotime). Proteins were separated by 4%–12% SurePAGE gels (GenScript, Nanjing, China), transferred to a nitrocellulose membrane (Pall, Mexico), and then detected using antibodies according to standard procedures. Primary antibodies were applied overnight at 4 °C for western blot tests, and their dilutions were as follows: NCAPG (sc-101014, Santa Cruz, 1:1000); MYHC (MF20, Developmental Studies Hybridoma Bank, 1:50); MYOD (sc-377460, Santa Cruz, 1:1000); MYOG (sc-12732, Santa Cruz, 1:1000) and β-tubulin (10094-1-AP, Proteintech, 1:2000). Finally, secondary antibodies were visualized with HRP-conjugated secondary antibodies that were applied for 1 h at room temperature. ECL western blotting detection reagent (Beyotime) was used to visualize the protein bands.

### 4.7. Lentivirus Infection

Cells were seeded in 3.5-cm culture dishes (Corning, Corning, NY, USA) with a 40%~50% confluence. A lentivirus expressing GFP and H2B was purchased from HanBio (Shanghai, China; www.hanbio.net). The lentivirus-coated GFP–H2B was transfected into cells with polybrene (HanBio). After 48 h of infection at 37 °C, the medium was replaced by fresh DMEM. The mitosis process was observed with a confocal microscope (TCS SP8, Leica, Wetzlar, Germany) at day 1.5.

### 4.8. TUNEL Staining

The apoptosis of myoblasts was examined using a TUNEL assay (Beyotime). The TUNEL assay was performed in accordance with the manufacturer’s protocols. TUNEL-positive cells (indicated by red fluorescent staining) were defined as having undergone apoptotic cell death. In terms of cell counts, TUNEL-positive cells were counted in three random fields of each section. The apoptosis index was calculated according to the following formula: the number of apoptotic cells/the total number of nucleated cells × 100%.

### 4.9. Caspase-3 Activity Detection

Caspase-3 activity was measured spectrophotometrically via the detection of pNA cleavage by caspase-3-specific substrates. These experiments were completed using a Caspase-3 Assay Kit (Beyotime). After the cell lysates were incubated with Ac-DEVD-pNA for 2 h at 37 °C, the samples were read at 405 nm.

### 4.10. ATAC-Seq and Data Analysis

The ATAC-seq libraries were constructed with a TruePrep DNA Library Prep Kit V2 for Illumina (Vazyme, Nanjing, China). Briefly, 50,000 collected cells (counted using trypan blue exclusion) were lysed in cold lysis buffer (10 mM Tris-HCl, pH 7.4, 10 mM NaCl, 3 mM MgCl2, 0.1% NP40, 0.1% Tween-20, and 0.01% *Digitalis* saponin) for 10 min on ice. After centrifugation at 500 g for 5 min, the nuclei were pelleted and resuspended in Transposase buffer. The transposition reaction was carried out for 30 min at 37 °C. Following purification, the libraries were amplified for 16 cycles and purified using VAHTS RNA Clean Beads (Vazyme). Libraries were quantitated using a Qubit 4 Fluorometer (Invitrogen, Singapore). Quality control of the libraries was performed with a Bioanalyzer 2100 (Agilent Technologies, Santa Clara, CA, USA, D1000 screentapes and reagents, 5067-5582). ATAC libraries were sequenced and multiplexed on an Illumina HiSeq X Ten with 150-bp paired ends. Raw sequence reads were initially processed for quality control by a FastQC (0.11.5), and then a Skewer (0.2.2) was used to remove the adapter sequences and poor-quality reads. Subsequently, the remaining reads were mapped onto the bovine reference genome of ARS-UCD1.2 using Burrows–Wheeler Alignment (BWA) (0.7.12). SAM files were converted into a BAM format using Samtools and were used for peak calling. A consensus map was created for each group by merging all samples using the BEDTools merge command. MACS2 (2.1.2) was used to call peaks. Correlations between libraries were calculated using the deepTools (3.0.2) bamCorrelate bins command. ATAC-seq peaks were annotated using a PeakAnnotator. A TF-binding motif analysis of ATAC-seq data was performed using HOMER (v4.9.1). Only known motifs from HOMER’s motif database were considered. We studied the motifs enriched by ATAC-seq peaks using findMotifsGenome.pl. A GO enrichment analysis of differential peaks was performed using the Goseq R package. GOseq was applied to assess enrichment, and topGO was used for plot directed acyclic graph bases of significantly enriched genes. We used KOBAS software (3.0) to determine whether differential peaks were significantly enriched in KEGG pathways. A motif enrichment analysis was performed with the findMotifsGenome.pl command in the HOMER package (4.9.1). The raw sequence data reported in this paper have been deposited in the Genome Sequence Archive (Genomics, Proteomics & Bioinformatics 2017) at the BIG Data Center (Nucleic Acids Res 2019), Beijing Institute of Genomics (BIG), Chinese Academy of Sciences, under accession numbers CRA002306 and CRA002306. They are publicly accessible at https://bigd.big.ac.cn/gsa.

### 4.11. Statistical Analyses

All data are presented as the mean ± the SEM from at least three independent experiments for each treatment. Data were analyzed using Student’s t-test and incorporated into GraphPad Prism version 6.0 software (GraphPad Inc., San Diego, CA, USA). *p* < 0.05 was considered to be statistically significant.

## Supplementary Materials

Supplementary materials can be found at https://www.mdpi.com/1422-0067/21/4/1248/s1.

## Abbreviations

NCAPG	Non-SMC condensin I complex subunit G
SMC	Structural maintenance of chromosomes
CAP-G	Chromosome-associated protein G
CAP-H	Non-SMC condensin I complex subunit H
CAP-D2	Non-SMC condensin I complex subunit D2
MYH	Myosin heavy chain
MYOG	Myogenin
MYOD	Myoblast determination protein 1
MYF5	Myogenic Factor 5
NEBD	Nuclear envelope breakdown
H2B	Histone H2B-like
IF	Immunofluorescence
KD	Knockdown
GFP	Green fluorescent protein
TUNEL	TdT-mediated dUTP nick-end labeling
H4K20me1	Histone 4 lysine 20 monomethylation
H4K16ac	Acetylation of histone H4 on lysine 16
H3K4me3	Histone H3 trimethylated at lysine 4
TSS	Transcription start site
KEGG	Kyoto Encyclopedia of Genes and Genomes
MAPK	Mitogen-activated protein kinase
NC	Negative control
MCK	Muscle creatine kinase
siRNA	Small interfering RNAs
ATAC-	Assay for Transposase-Accessible Chromatin
seq	High-throughput sequencing
TSS	Transcription start sites
AP-1	Activating protein 1
c-Jun	Transcription factor AP-1-like
JUNB	JunB proto-oncogene
JUND	JunD proto-oncogene
FOSL2	Fos-related antigen 2
FOSL1	Fos-related antigen 1
PRKAA1	Protein kinase AMP-activated catalytic subunit alpha 1
SIRT1	Sirtuin 1
CTCF	CCCTC-Binding Factor
BATF	Basic Leucine Zipper ATF-Like Transcription Factor
TBP	TATA-binding protein
PP2A	Protein phosphatase 2A
DAPI	4′,6-diamidino-2-phenylindole
