# Supplementary material for: NCAPG Dynamically Coordinates the Myogenesis of Fetal Bovine Tissue by Adjusting Chromatin Accessibility
Source: Int J Mol Sci. 2020 Feb 13;21(4):1248. doi: 10.3390/ijms21041248 (PMC7072915; doi:10.3390/ijms21041248)
Supplement: Supplementary file 1 [file ijms-21-01248-s001.zip › Supplementary Tables.docx]

**Supplementary Table 1.** Overview of raw data output and quality assessment.

| **Sample** | **Raw_reads** | **Clean_reads** | **Clean_rate** | **Mapped_MT/PT** | **Mapped_non_MT/PT** | **Unique_mapped** | **Unique_mapped_dedup** |
| --- | --- | --- | --- | --- | --- | --- | --- |
| NC1 | 64,702,355 | 63,017,485 | 97.40% | 693,935(1.10%) | 61,012,019(96.82%) | 51,554,083(81.81%) | 38,692,482(61.40%) |
| NC2 | 73,660,084 | 70,825,570 | 96.15% | 691,759(0.98%) | 69,080,399(97.54%) | 57,624,571(81.36%) | 43,200,268(61.00%) |
| si*NCAPG*1 | 66,482,524 | 63,660,318 | 95.75% | 665,727(1.05%) | 62,020,858(97.42%) | 49,204,438(77.29%) | 36,765,466(57.75%) |
| si*NCAPG*2 | 67,116,879 | 63,413,268 | 94.48% | 673,688(1.06%) | 61,350,301(96.75%) | 46,965,337(74.06%) | 36,506,294(57.57%) |

**Supplementary Table 2.** Overview of the ATAC-seq peak quantification.

| **Sample** | **Count_of_narrow_peak** | **FRiP** | **Count_of_summits** |
| --- | --- | --- | --- |
| NC1 | 110,756 | 0.268369 | 134,862 |
| NC2 | 115,989 | 0.272941 | 140,187 |
| si*NCAPG*1 | 74,896 | 0.157104 | 87,725 |
| si*NCAPG*2 | 71,117 | 0.13742 | 80,608 |

**Supplementary Table 3.** Sequences of siRNA.

| **Gene Name** | **siRNA** | **Sequence (5’-3’)** |
| --- | --- | --- |
|  | siRNA001 | CCAGCTGTGGAAAGAGTAA |
| *NCAPG* | siRNA002 | CCACACCAATATCCCTAAT |
|  | siRNA003 | GCACAGGATGACATCACAA |

**Supplementary Table 4.** Primers for real-time quantitative PCR.

| **Gene** | **Sequence (5’-3’)** | |
| --- | --- | --- |
| *NCAPG*  *NCAPH*  *SMC2*  *SMC4*  *NCAPD2*  *NCAPD3*  *NCAPG2*  *NCAPH2* | Forward | CTTTCCGATTTCTTAGATAGTGAG |
|  | Reverse  Forward  Reverse  Forward  Reverse  Forward  Reverse  Forward  Reverse  Forward  Reverse  Forward  Reverse  Forward  Reverse | AAACATTCCTGGTTAGTCCTG  TCGGCCTGCACTTGATTGA  GCCTTCTTGGCTGTTCCTGTT  AGGCAGGTAGTTATTGGTGGCA  GCATGATGAGAAAGTGAGGGTTG  TTACTTTGGGAGGTGATGCTGA  GCAAACACTAAAGCCAATGAACTAA  GGGGTGAATCAGTATGTCGTGC  TTCCTTGAGGCCAGGGTCTAT  GCGGTTCAACATCACTTCCAG  AGCTTCTTCTGCGACTCCTGC  TGTGACCCGTTCAACCTAAATG  TGACACAGAAGCAAGAATCACAGA  TCGTCAGTGGGACCCTCATC  CGTGTTCACCCTGAAATCCTT |
| *MYOG* | Forward | CCGTGGGCGTGTAAGGTGTG |
|  | Reverse | CCTCTGGTTGGGGTTGAGCAG |
| *MYOD* | Forward | TTTGCCAGAGCAGGAGCCCCTC |
|  | Reverse | TTCGAACACCTGAGCGAGCGC |
| *MYF5* | Forward | TGGCTGCTTTCGGGGCTCAC |
|  | Reverse | GGTTGACCTTCTTCAGGCGTCTCC |
| *MYH1* | Forward | GGGAAACTGGCTTCTGCTGAT |
|  | Reverse | TGGGTTGGTGGTGATTAGGAG |
| *MYH2* | Forward | GTCAAAGGGACTATCCAGAGCAG |
|  | Reverse | AGAAGAGGCCCGAGTAGGTGT |
| *MYH3* | Forward | TAAGGAAGAGTATGCCAAGGGG |
|  | Reverse | CATCCAGGAGGTGTAGCGGTC |
| *MYH4* | Forward | CTCCTAATCACCACCAACCCATA |
|  | Reverse | TGTCAGCAACTTCAGTGCCATC |
| *FOSL2* | Forward | CGGTGTGATCAAGACCATTG |
|  | Reverse | CTTCTCCTCCTCCAGCTCCT |
| *JUND* | Forward | CTCAAGGATGAACCGCAGAC |
|  | Reverse | GGCTCTTGAGCGTCTTCACT |
| *JUNB* | Forward | TACCACGACGACTCATACGC |
|  | Reverse | CCTGGCTGGAAAAGTAGCTG |
| *JUN* | Forward | AAAACCTTGAAAGCGCAGAA |
|  | Reverse | TCTGTTTCCCTCTCGCAACT |
| *18S* | Forward | GTAACCCGTTGAACCCCATT |
|  | Reverse | CCATCCAATCGGTAGTAGCG |
