# Supplementary figures and images for: NCAPG Dynamically Coordinates the Myogenesis of Fetal Bovine Tissue by Adjusting Chromatin Accessibility
Source: Int J Mol Sci. 2020 Feb 13;21(4):1248. doi: 10.3390/ijms21041248 (PMC7072915; doi:10.3390/ijms21041248)

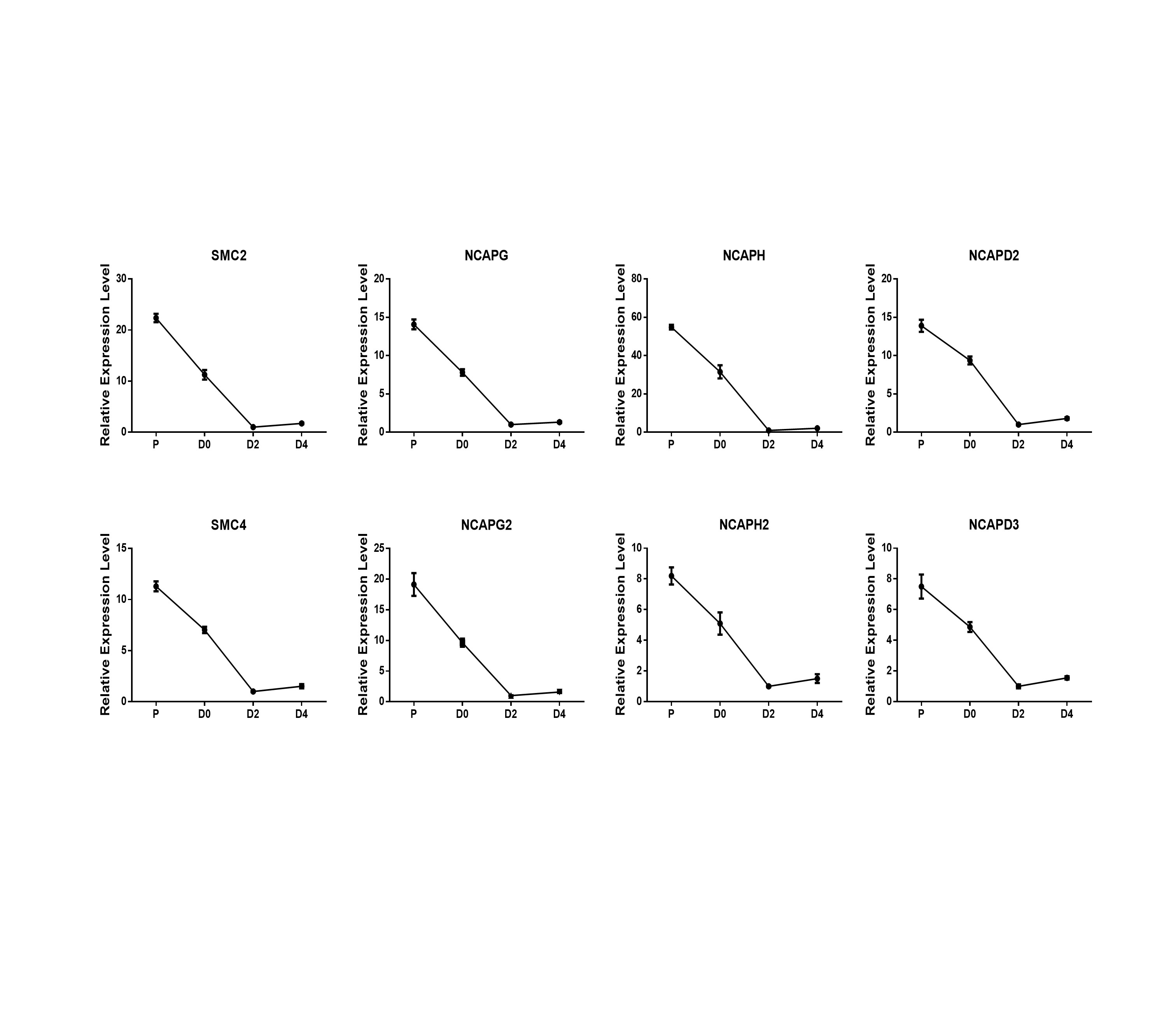

Supplement: Supplementary file 1 [file ijms-21-01248-s001.zip › Fig.S1.JPG]

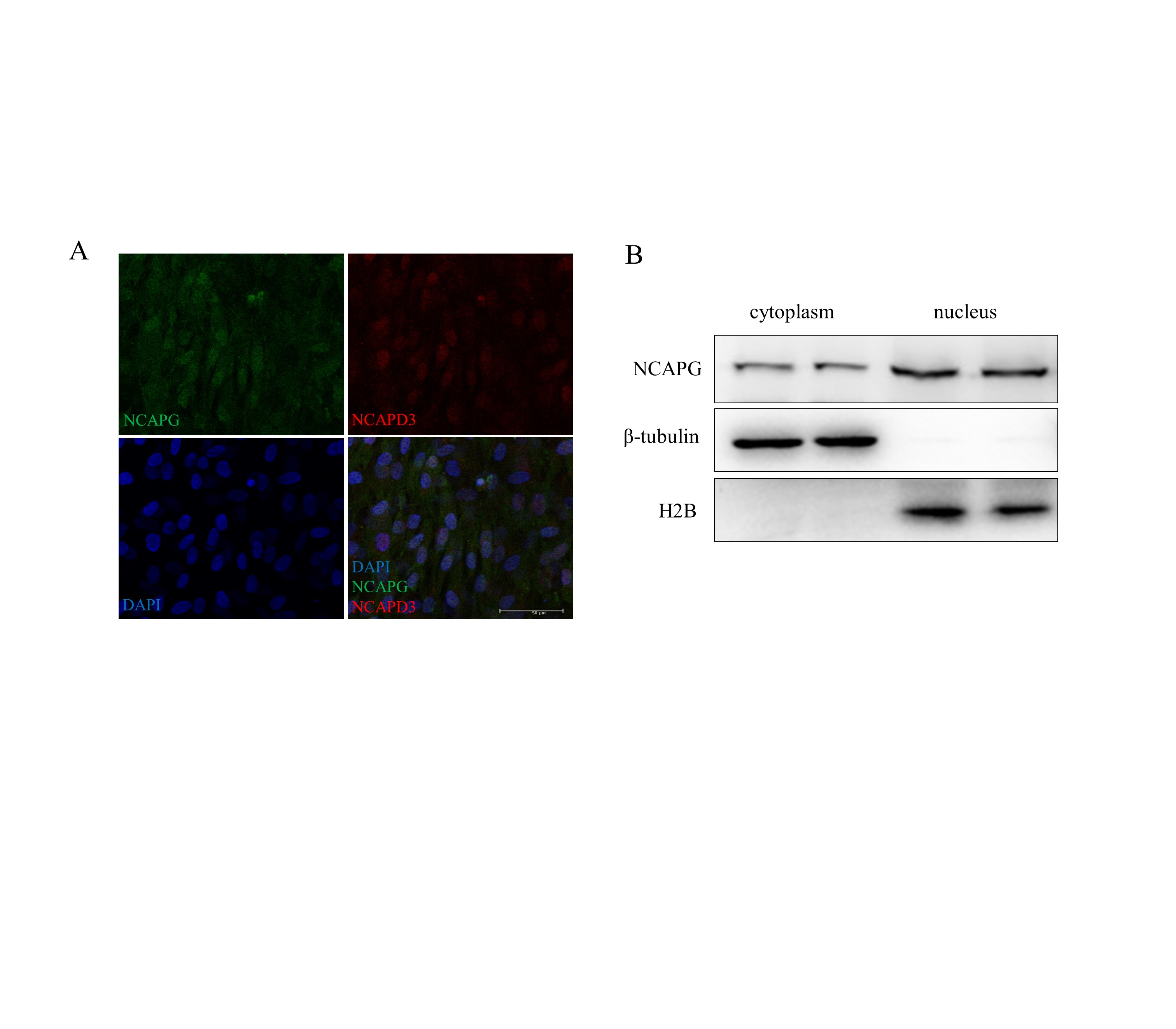

Supplement: Supplementary file 1 [file ijms-21-01248-s001.zip › Fig.S2.JPG]

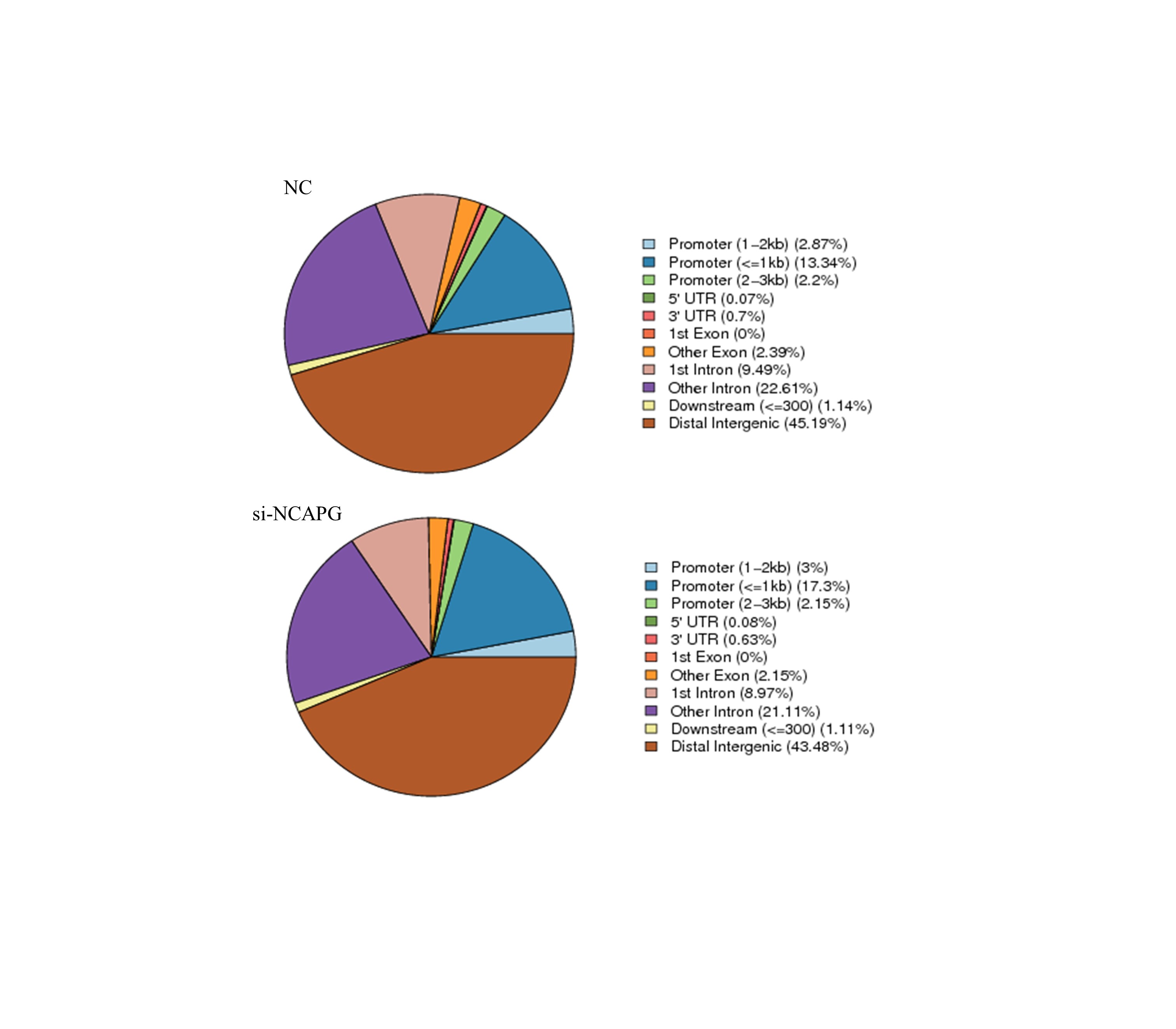

Supplement: Supplementary file 1 [file ijms-21-01248-s001.zip › Fig.S3.JPG]

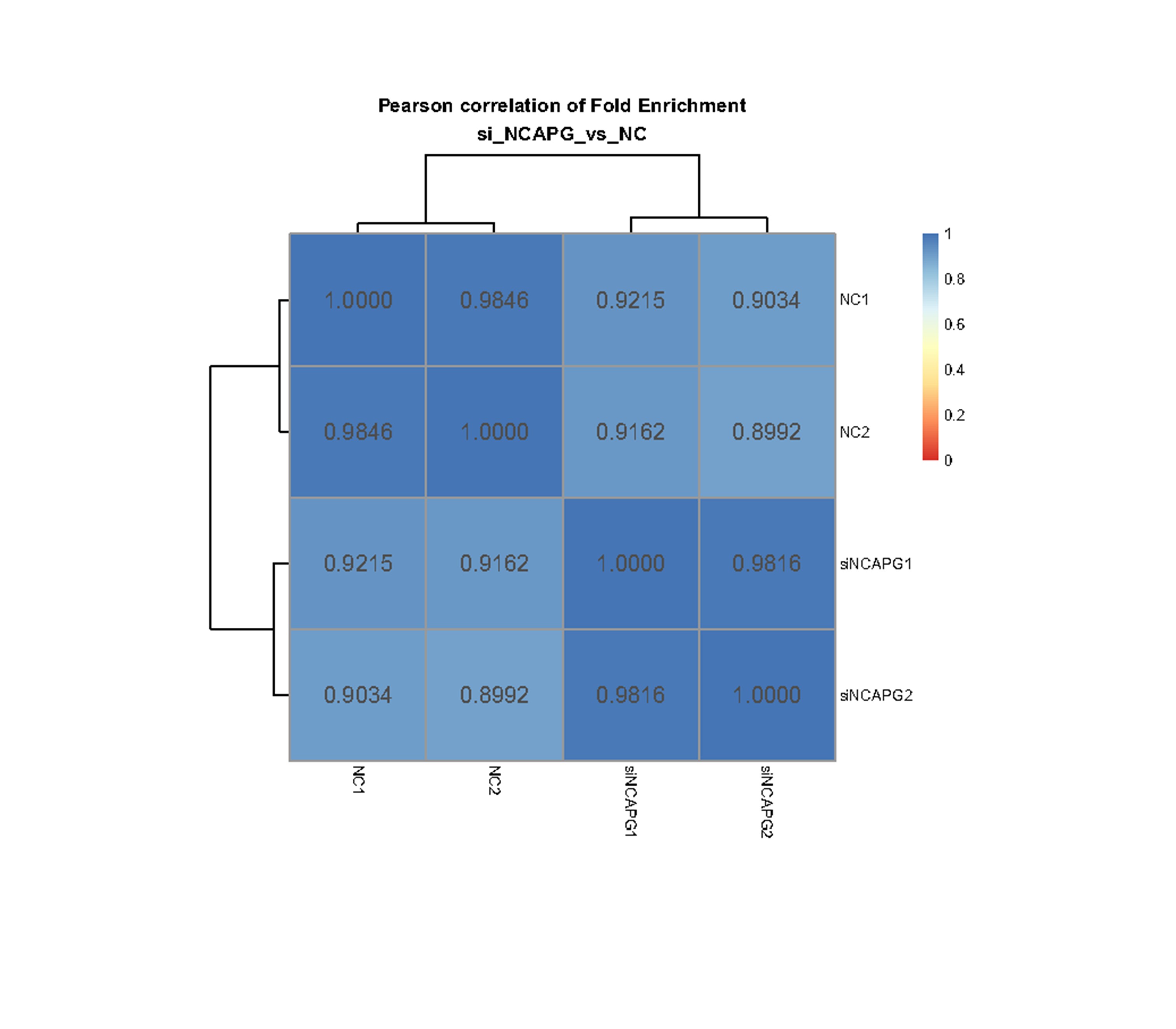

Supplement: Supplementary file 1 [file ijms-21-01248-s001.zip › Fig.S4.JPG]

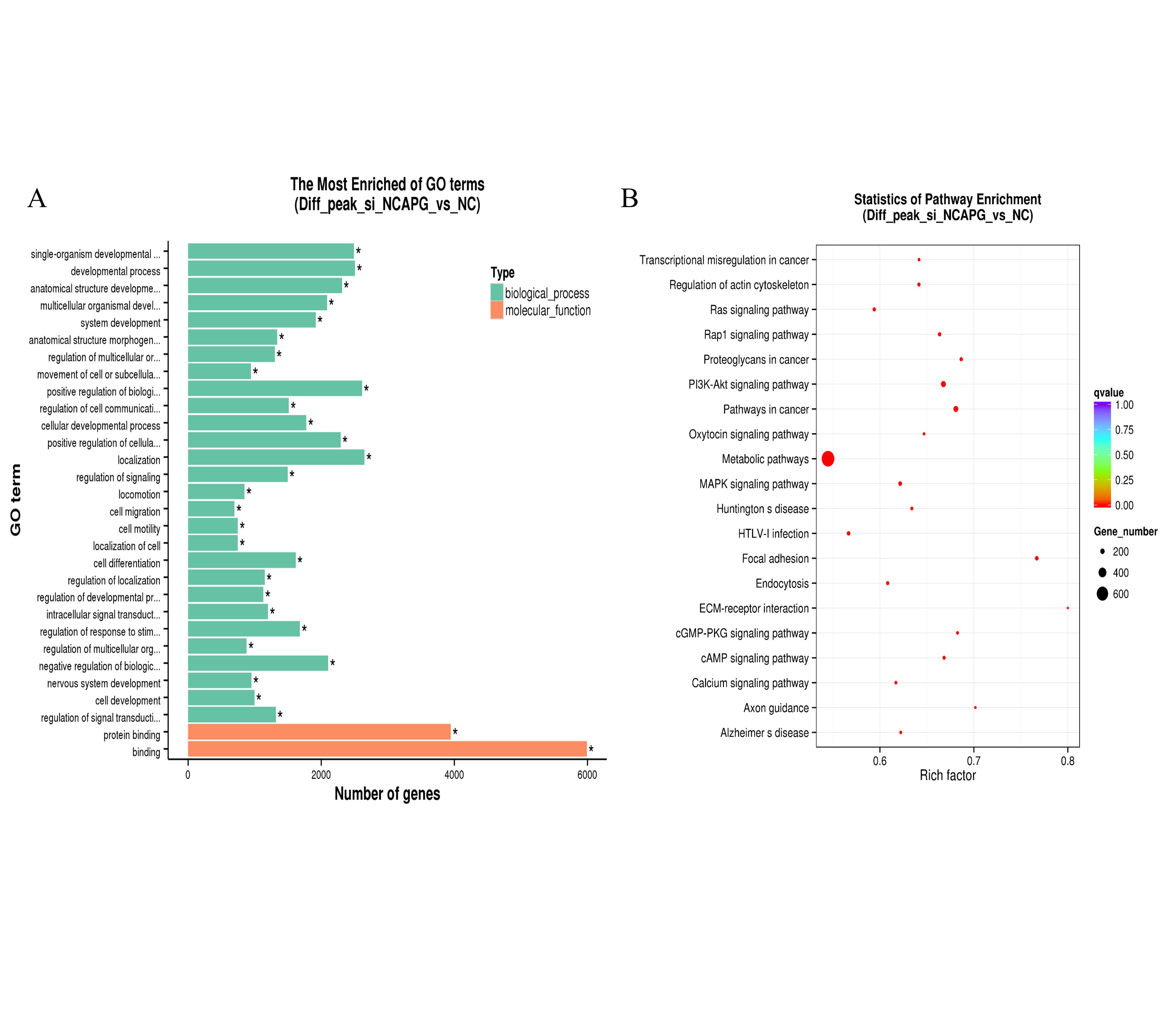

Supplement: Supplementary file 1 [file ijms-21-01248-s001.zip › Fig.S5.JPG]

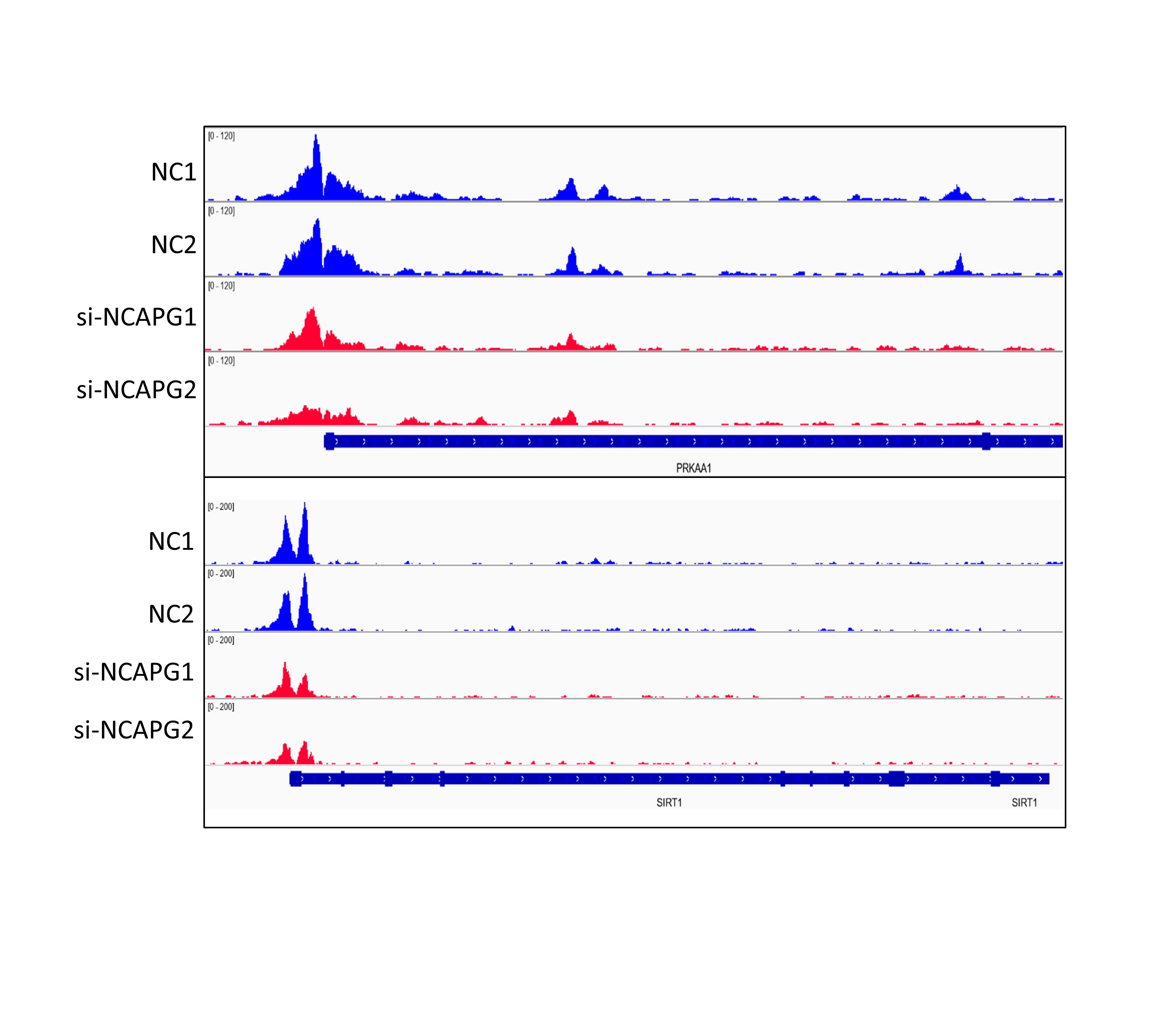

Supplement: Supplementary file 1 [file ijms-21-01248-s001.zip › Fig.S6.JPG]
